# The CDC/APHL next generation sequencing quality initiative: practical guidance to implementing quality management systems in laboratories performing next generation sequencing (phase 2)

**DOI:** 10.1128/jcm.00213-26

**Published:** 2026-07-20

**Authors:** Blake Cherney, Ariel Diaz, Christopher Ghattas, Mia Gregory, Emily Stow, Jennifer Onyewuenyi, Lakshmi Soujanya Kocherlakota, Lucy White, Eshaw Vidyaprakash, Sarah Scott, Jeffrey K. Lamar, Glenita McKinney, Urmil Parekh, Diego Arambula, Heather Stang, On behalf of the CDC/APHL NGS Quality Initiative

**Affiliations:** 1 Office of Laboratory Systems and Response, Division of Laboratory Systems, Centers for Disease Control and Prevention 1242, Atlanta, Georgia, USA; 2 Booz Allen Hamilton, Atlanta, Georgia, USA; Medical College of Wisconsin, Milwaukee, Wisconsin, USA

**Keywords:** next generation sequencing, quality management system, Quality System Essentials, public health laboratories, regulatory, clinical laboratory, quality assurance, quality control, method validation, Clinical and Laboratory Standards Institute

## Abstract

Whenever next generation sequencing is used in clinical and public health laboratories, rigorous quality control and quality assurance are key components of a quality management system. However, both the development and maintenance of a quality management system encompassing next generation sequencing present significant challenges, such as selection of appropriate sequencing platforms, complex workflows, allocation of time and resources, kit and reagent management, potential discontinuation of technical support, specialized bioinformatic workflows, compliance with regulatory and/or accreditation bodies, and the necessity of highly trained personnel. In an effort to address such challenges, the Centers for Disease Control and Prevention collaborated with public health partners to establish the Next Generation Sequencing Quality Initiative, which focuses on the development of a sequencing-focused quality management system. Here, we describe the ongoing efforts of the Next Generation Sequencing Quality Initiative to develop a foundational quality management system specific to next generation sequencing, as well as collaborations aimed to assist partners in addressing ongoing and emerging quality-related challenges associated with assay implementation in laboratories for surveillance and diagnostic applications.

## INTRODUCTION

Next generation sequencing (NGS) produces data ranging from hundreds of kilobases to multiple terabases within a single run. These data promote rapid diagnoses, accurate monitoring, and the detection of multiple agents simultaneously (1–6). Laboratories performing NGS likely benefit from the implementation of a quality management system (QMS) with quality assurance (QA) and quality control (QC) processes and procedures that facilitate method validation, records management, data management, and reporting of test results. However, establishing a laboratory QMS encompassing NGS presents several key challenges due to the inherent complexity of workflows, as well as barriers related to cost, method validation, specialized QA and QC processes, training of personnel, and equipment management (1, 2, 7, 8).

What makes NGS difficult from a quality management standpoint is the dynamic nature of its components and workflows. For example, frequent updates to sequencing chemistries and software require robust change control plans that can accommodate these rapid and sometimes abrupt modifications without compromising assay performance. Keeping pace with these updates is challenging, as implementation and oversight require subject matter experts with up-to-date knowledge of NGS, regulatory compliance, and safety considerations. Additionally, the validation of bioinformatic pipelines presents unique challenges, as these computational workflows evolve independently of wet-bench processes and require continuous verification to ensure accuracy and reproducibility (9). Furthermore, the governance and curation of reference databases used for sequence interpretation are critical, as inconsistencies or outdated information can lead to misinterpretation of results. These factors necessitate a QMS that is flexible, comprehensive, and specifically tailored to address the evolving landscape of NGS technologies and applications.

An effective QMS requires a systematic approach to quality practices and should describe, document, implement, evaluate, and monitor laboratory operations to provide high-quality services and comply with regulatory standards (10, 11). One QMS framework widely utilized by clinical and public health laboratories (PHLs) is A Quality Management System Model for Laboratory Services (QMS01) from the Clinical and Laboratory Standards Institute (CLSI). The CLSI’s framework utilizes 12 Quality System Essentials (QSEs), which serve as the quality building blocks for an end-to-end workflow that aligns with standard QMS elements and regulatory requirements (e.g., the Clinical Laboratory Improvement Amendments of 1988 [CLIA], ISO 15189 international standard) (10–12).

**Fig 1:**
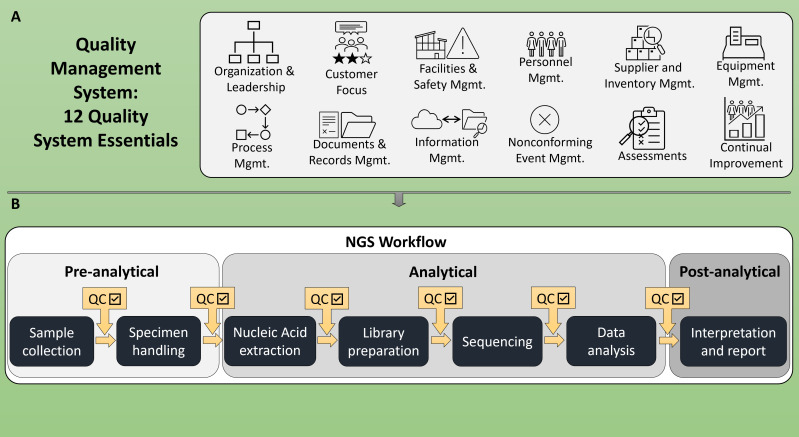
Flowchart quality management system for an NGS laboratory. (**A**) The Clinical and Laboratory Standards Institute’s 12 Quality System Essentials act as building blocks for laboratories establishing a quality management system. (**B**) A general NGS laboratory workflow encompassing pre-analytical (sample information and collection and sample handling), analytical (nucleic acid extraction, library preparation, sequencing, and data analysis), and post-analytical stages (interpretation and report) is regulated by quality control (QC) checkpoints.

The implementation of NGS into research, surveillance, and clinical settings has revealed challenges associated with establishing assays, many of which have led to the development of best practices as well as consensus documents; nevertheless, the need for additional, prescriptive, regulatory guidelines remains (5). To better support laboratories performing NGS in an ever-changing landscape, the Centers for Disease Control and Prevention (CDC) worked with partner institutions to apply QMS and NGS guidelines to create tools and resources that can assist laboratories in developing NGS workflows (1, 2, 4, 5, 7, 9, 13–15). NGS techniques are constantly advancing, with improvements in automation and sequencing chemistries driving increased throughput and better performance (3, 13). Individual laboratories develop sequencing assays utilizing individualized standards and guidelines; however, the need remains to align QC and QA across NGS platforms, applications, agents of interest, and laboratories to advance public health and clinical practices. A QMS with defined QC checkpoints, QA, and continuous monitoring during all phases of the laboratory workflow can provide NGS results that improve research, surveillance, and clinical applications by translating reliable, accurate data to expand the understanding of health issues, support public health and clinical decisions, and improve patient outcomes (Fig. 1).

## THE CDC/ASSOCIATION OF PUBLIC HEALTH LABORATORIES NGS QUALITY INITIATIVE

The CDC, the Association of Public Health Laboratories (APHL), and state and local PHLs collaborated to form the NGS Quality Initiative, which assists clinical and PHLs in developing an NGS-focused QMS. The Initiative brought together public health leaders, laboratory scientists, microbiologists, bioinformaticians, quality managers, and other subject matter experts for a consensus-based approach to identify challenges in establishing and maintaining NGS assays in clinical and PHLs.

**Fig 2:**
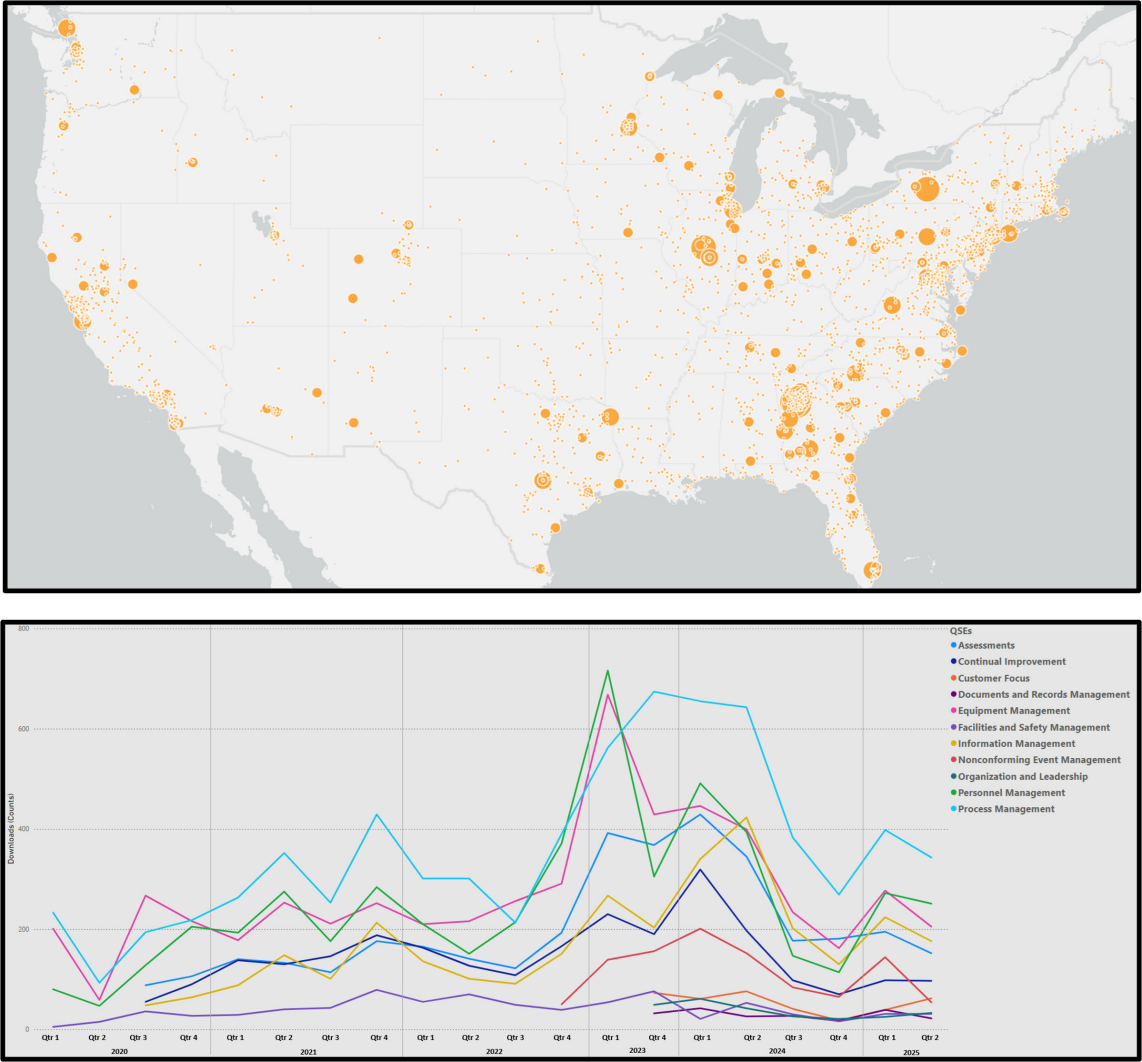
NGS Quality Initiative website traffic. (Top) Geographic distribution of U.S. visitors from 2020 (quarter 1) to 2025 (quarter 2); visitors from Alaska and Hawaii are not displayed, but visitors by state are reported in Table S1. (Bottom) Total downloads (counts, *Y* axis) per Quality System Essential (QSE) and per quarter (Qtr, *X* axis). All QSEs are listed in the legend, and each dot represents the total document downloads for the specific quarter and QSE.

Using CLSI’s 12 QSEs as a guide, the NGS Quality Initiative works to develop and publish customizable tools, resources, standard operating procedures (SOPs), forms, and logs, among other products, which can be used as a foundational system or integrated into a laboratory’s existing QMS (9). To promote open access to its tools, the NGS Quality Initiative developed a publicly accessible website that contains NGS-related information and all its QMS resources (https://www.cdc.gov/lab-quality/php/ngs-quality-initiative/; Supplemental materials). Since 2019, the Initiative has published 115 documents (Table 1) and several tools. From 2020 to 2025, NGS Quality Initiative products have been downloaded approximately 29,000 times, with roughly 48,000 unique visitors to the website (Fig. 2; Table S1), and information gathered through various channels (e.g., emails, surveys, and workgroups [data not shown]) indicates many laboratory personnel are interested in using the Initiative’s resources (8, 9, 14). The NGS Quality Initiative builds on previously published work, consensus guidelines, and best practices (when possible) to develop an expanded framework of resources to further laboratory implementation of quality practices for NGS workflows under research, surveillance, and diagnostic applications (14).

**TABLE 1:** Quality System Essentials and NGS Quality Initiative resources

Challenge	Risk	Documents addressing challenge^a^
QSE: Personnel Management		
Time commitment and resources to train personnel are required.	Staff who are not fully trained and proficient are more likely to make errors.	1–25
A mismatch between existing skill sets and needed skill sets in personnel who will perform NGS assays may be present.	Inexperienced staff will have difficulty troubleshooting NGS processes.	1–25
A formal training and assessment system for personnel working with high-complexity testing, such as bioinformatics and information technologists, is needed.	Laboratories without bioinformatic expertise might use computer resources inefficiently and fail audits due to undocumented and informal trainings.	1–3, 22–25
QSE: Equipment Management		
NGS equipment may require accommodation to ensure physical environment requirements are met (e.g., minimal vibration, specific temperature, and humidity ranges).	Laboratories not aware of equipment requirements before instrument arrival face delays and unplanned costs.	39, 44–47, 50, 54, 60
NGS equipment requires installation qualification, operational qualification, and performance qualification. Additionally, it might require regular manufacturer- or user-performed preventive maintenance or re-qualification.	If qualifications and maintenance are not performed according to schedule and established processes, the sequencing results may be compromised.	28–33, 35–38, 42–43, 45–46, 48–49, 51–53, 57–59, 61
Frequent updates are released for NGS hardware and software, requiring verification of method performance or even revalidation.	Frequent verifications and validations require substantial time and are costly; laboratories risk out-of-control processes/results if the updates are not tracked and documented.	40–41, 45, 55–56
A variety of sequencing platforms may be available, and information to help with the selection is needed.	Personnel might not select the most appropriate equipment according to testing needs (e.g., short reads, long reads, and easy setup).	39, 44, 46–47, 50, 54, 60
QSE: Process Management		
There are several different processes that comprise the overall NGS workflow.	An error early in the process can result in the use of expensive reagents downstream and poor-quality sequence data outputs.	62–63, 66, 68–70
The NGS workflow uses multiple sets of reagents at different cost points.	Inappropriate execution of validation and verification could result in a waste of resources and time.	64, 66, 73
NGS-based assays require a more complex and time-consuming validation and verification than conventional assays.	Incomplete understanding of assay performance can translate into the reporting of inaccurate results.	64–67
Bioinformatic analysis requires the time of highly skilled personnel and computational resources.	Poor-quality sequence data fed into the analysis pipeline may consume computing resources and personnel time, thereby increasing unnecessary resource expenditures.	62–66, 71–73
QSE: Information Management		
Documenting bioinformatic tools used to analyze samples	Without the ability to track samples and associated results, there is a risk of providing inaccurate information, impacting decision-making and patient outcomes.	75–87
Collaborating to share bioinformatic pipelines with partner laboratories	Misunderstanding and miscommunication of bioinformatic methods can lead to divergent results for the same data set.	75, 79–81, 86
Storing and retrieving patient data using an appropriate file format	Storing and using inappropriate types of files can lead to unexpected cloud storage expenses, information loss, and low computer performance.	76–81, 84–87
QSE: Assessments		
Laboratory leadership is responsible for assessing the accuracy of new methods, and it can be difficult to determine key metrics to measure performance.	If metrics chosen to represent the accuracy of the system are not indicative of performance, the system may be noncompliant without the knowledge of laboratory leadership.	88–91
Overall QMS assessment of an NGS laboratory	Laboratory personnel might fail to identify weaknesses in the current QMS framework. Altogether, this could translate into audit failures and invalid test results.	91
QSE: Continual Improvement		
Selecting the most impactful areas for improvement in a complex system can be difficult.	Selecting areas for improvement that do not provide the highest impact risks misaligning laboratory resources with the need for high-quality reporting of accurate results.	92–93
QSE: Facilities and Safety Management		
Assessing the biological and chemical risk of various components in a complex process is time-consuming.	Improper disposal of laboratory waste can lead to health and environmental risks.	94–98
QSE: Supplier and Inventory Management		
Forecasting supplies and inventory properly to meet the laboratory’s needs is required.	Laboratory management might fail to predict essential supplies to produce test results. This may result in increased turnaround time of patient results and a waste of resources.	99–101
A system for tracking the location and use of samples and reagents is needed.	Laboratories might use unacceptable samples and expired reagents, which can result in invalid test results.	99–101
QSE: Documents and Records Management		
Selecting an approach to data management and retention of large NGS data sets that will meet the requirements while also being cost-effective	Failing to retain key data can lead to loss of decision-making accuracy, and storage costs can quickly add up, thereby making NGS cost prohibitive.	102
Updating and documenting the version control of laboratory procedures	Using outdated SOPs due to manufacturers’ changes might lead to inaccurate test results.	102
QSE: Nonconforming Event Management		
Establishing an adequate process to track out-of-compliance events, find the root cause, and action items for future actions	Failing to report and correct failures can lead to failed audits and inaccurate results; it can also impact patient health outcomes.	103–108
QSE: Organization and Leadership		
Establishing a communication plan and responsibilities	Failing to communicate may lead to expired instrument maintenance, as well as inefficient wet- and dry-laboratory workflow coordination.	109–111
QSE: Customer Focus		
Effectively and properly reporting NGS data to customers	Failing to provide specific information in the test result report may lead to inaccurate diagnostic results and poor patient outcomes.	113–114
Improving a QMS based on customers’ feedback	Failing to provide timely information regarding test results may result in customer dissatisfaction, loss of credibility, legal exposure, and regulatory noncompliance, and it could impact patient outcomes and quality of life.	113–114

^a^ Numbers third column are referencing documents Table S1.

Outlined here by QSE, we provide brief descriptions of associated challenges and resources (i.e., NGS Quality Initiative’s published products) that help to mitigate the impact of such challenges (Table 1).

## QSE: PERSONNEL MANAGEMENT

This QSE describes requirements for onboarding and retaining qualified, trained, and competent personnel who perform and manage laboratory activities (9, 11, 14). Personnel Management is vital to the laboratory; this QSE ensures that staff possess the requisite qualifications, undergo comprehensive training, demonstrate competency, exhibit the ability to perform assigned tasks proficiently, actively engage in continuous improvement, and implement and maintain the QMS effectively. The complexity of NGS workflows poses various challenges related to Personnel Management, including the need for specialized NGS training and assessments, the variety of sequencing platforms and associated equipment, specialized training to build and run bioinformatic pipelines, and the need to comply with testing regulations (e.g., CLIA regulations, or applicable regulatory and accreditation requirements) (10, 13). Proof of competency is also required, e.g., a biochemist operating an NGS assay must have demonstrated proficiency operating that assay under direct supervision (14). To overcome technical and quality-related challenges related to this QSE, the NGS Quality Initiative has published 25 products specific to laboratory personnel, including training procedures, sequencer-specific competency assessments, and training forms (Table 1; Table S1 [documents 1–25]). Since 2020, there have been over 4,000 downloads of Personnel Management-related resources (Fig. 2B; Table S3). For instance, since training bioinformatics personnel via a consistent methodology remains a challenge for NGS laboratories, the NGS QI developed the Bioinformatics Employee Training SOP, which outlines a proper sequence of training and criteria for the demonstration of competency.

## QSE: EQUIPMENT MANAGEMENT

Equipment Management is critical for ensuring that laboratory instruments function according to established criteria; effective management includes robust preventive maintenance and calibration programs as well as proper documentation of equipment-related problems (11, 14). Equipment used for NGS can be highly specialized, requiring detailed knowledge of proprietary engineering and associated software to troubleshoot and repair (7, 15). Additionally, laboratories performing testing under CLIA require procedures that ensure adherence to manufacturers’ recommendations for both hardware and software, or applicable regulatory and accreditation requirements (10). To reduce the impact and risks associated with NGS equipment, the NGS Quality Initiative published 36 equipment-related, customizable tools, resources, and SOPs (Table 1; Table S1 [documents 26–61]). Since 2020, there have been over 4,000 downloads of Equipment Management resources (Fig. 2B; Table S2). The three most downloaded tools for equipment are the pre-installation checklists for MiSeq, iSeq 100, and MinION Mk1B sequencers, the most popular platforms in public health settings. These tools address some of the most consistent challenges for laboratories: equipment selection, installation preparation, maintenance, and recordkeeping.

## QSE: PROCESS MANAGEMENT

Process Management refers to all laboratory activities and phases associated with the testing process (i.e., pre-analytical, analytical, and post-analytical stages) and is crucial to the quality of the laboratory’s result reporting, which dictates the impact and value of testing results for the target audience and/or customer(s) (11, 14, 16). Incorporating NGS workflows into this QSE can be complex due to the lack of experienced personnel who, e.g., may need to prepare a method validation or establish a QC plan (8, 17–21). Process Management is further complicated by frequent technical modifications, which are often required due to updates in software, sequencing platforms, and bioinformatic analysis tools. In addition, turnover in kit chemistries can create significant challenges, as changes to kit chemistries can occur frequently, sometimes in as few as 2 years. Change control plans should be built into method validation plans, especially since these changes can happen abruptly and frequently. Failure to properly manage workflow activities may result in adverse outcomes and other roadblocks. Laboratories performing testing under CLIA are required to demonstrate reproducible results and assay performance according to specifications defined by the laboratory or manufacturer, which highlights the need for uniformity in laboratory processes or applicable regulatory and accreditation requirements (10). To reduce risks associated with this QSE, the NGS Quality Initiative published 12 customizable resources (Table 1; Table S1 [documents 62–73]). Since 2020, there have been over 7,000 downloads of resources related to Process Management (Fig. 2B; Table S2). The three most downloaded products help mitigate a common challenge for NGS laboratories: method validation. These products are the NGS plan template, SOP, and summary report, all of which support the validation of NGS laboratory-developed tests.

## QSE: INFORMATION MANAGEMENT

Information Management encompasses those processes necessary for proper data management. It offers guidance on managing and accessing information, including records entered into laboratory notebooks or electronic record management systems (11). Laboratories performing NGS manage extensive amounts of data while ensuring data accuracy, validity, completeness, integrity, timeliness, and immutability (3, 22). Laboratories need policies, processes, and procedures that address data and information access. Furthermore, security plays an important role in Information Management, especially under CLIA regulations, where confidentiality and data integrity are of the utmost importance during transmission of patient data or applicable regulatory and accreditation requirements (10). To assist laboratories performing NGS-based assays, the NGS Quality Initiative has developed 14 customizable Information Management tools (Table 1; Table S1 [documents 74–87]). Since 2020, there have been over 3,000 downloads of Information Management resources (Fig. 2B; Table S2). The three most downloaded documents are Sequencing Data Storage and Retention Best Practices, Bioinformatic Pipeline Documentation Standardization—Technical Best Practices, and Bioinformatic Pipeline Documentation Management–Laboratory Management Briefing. These documents ensure consistent and accurate laboratory workflows and serve as references for laboratories, thereby standardizing procedures, reducing errors, and enhancing overall productivity. Information Management resources also provide support for bioinformatic pipeline documentation, containerization of computer resources, and practices for data sharing, storage, and retention, among other NGS-related applications.

## QSE: ASSESSMENTS

Internal and external laboratory assessments are essential to verify that all processes meet performance and regulatory requirements (9, 11). An internal audit allows the laboratory to evaluate its processes. In contrast, external audits are conducted by entities external to the laboratory, like regulatory bodies or accrediting organizations (e.g., Centers for Medicare & Medicaid Services and the College of American Pathologists, respectively) (23). Failure to meet the requirements of any such auditing organization may result in the suspension or termination of laboratory services until deficiencies have been resolved. Due to the complexity of NGS workflows, assessments often require specialized skills and expertise in evaluating wet- and dry-bench workflows. To assist NGS laboratories, the NGS Quality Initiative has developed five customizable assessment tools (Table 1; Table S1 [documents 88–91]). These tools help evaluate laboratories’ QMS frameworks and aid in assessing bioinformatic pipelines. Because evaluating an entire QMS can be difficult, the NGS QI developed the QMS Assessment Tool, which serves as a valuable resource for users to assess their laboratory’s QMS (with a focus on NGS framework) to generate qualitative and quantitative data. The QMS Assessment Tool provides laboratories with comprehensive question sets that, once answered, produce weighted scores related to each of the 12 QSEs. This allows users to evaluate laboratory quality practices and identify gaps and areas for improvement while tracking compliance over time (e.g., by quarter, by QSE). Since 2020, there have been more than 3,500 downloads of resources related to the QSE Assessments (Fig. 2B; Table S2), approximately 2,500 of which were downloads of the QMS Assessment Tool.

## QSE: CONTINUAL IMPROVEMENT

The QSE Continual Improvement defines a strategy and provides information for identifying opportunities to improve laboratory processes (10, 12). Because laboratories constantly refine their workflows and methods, continual improvement is paramount for risk mitigation and risk management. Without a laboratory policy that is strongly aligned with the principles of continual improvement, organizations may struggle to implement and maintain successful risk management programs. Further care must also be taken in standardizing processes for validation of NGS assays.

NGS test systems are divided into wet- and dry-laboratory workflows (e.g., nucleic acid, library preparation, and bioinformatic analysis; Fig. 1). In addition, each step requires specific equipment, protocols, and QC metrics. Such requirements can be challenging, especially as laboratories work to implement adequate monitoring and improvement processes that ensure compliance with regulations, such as those defined by CLIA, or applicable regulatory and accreditation requirements (11). To address these challenges, the NGS Quality Initiative developed two tools, which provide a framework for setting goals and identifying, monitoring, and assessing progress (Table 1; Table S1 [documents 92 and 93]). One of the tools, an SOP entitled Identifying and Monitoring NGS Key Performance Indicators, facilitates the monitoring of test performance, laboratory processes, and potential test system drift through metric tracking and evaluation. Since 2020, there have been over 2,000 downloads of Continual Improvement resources (Fig. 2B; Table S2).

## QSE: FACILITIES AND SAFETY MANAGEMENT

Laboratory facilities must meet physical requirements and established processes and procedures that ensure personnel safety and that provide proper environmental conditions (11). This includes providing information about the laboratory’s physical environment and implementing the facility maintenance and safety programs needed to support the laboratory and its activities (9, 24). Due to the nature of some NGS workflows, laboratory personnel face potential risks. For example, chemical exposure involving sequencing reagents (e.g., formamide and phenol) can be toxic in nature. Plans to mitigate and handle such risks and exposures are critical, including training to understand and follow safety data sheets (e.g., how to use appropriate personal protective equipment). Additional considerations include risk assessments and near-miss events; potential equipment failures in high-containment laboratories present an added layer of complexity due to access being restricted to specialized personnel. To assist laboratories with such challenges, the NGS Quality Initiative has created five customizable waste disposal documents for Illumina and Oxford Nanopore instruments (Table 1; Table S1 [documents 94–98]), including the Oxford Nanopore Flow Cell Reuse or Return SOP, which clarifies reuse, return, and disposal methods for flow cells, processes that are critical but sometimes convoluted or confusing. Since 2020, there have been over 700 downloads of QSE Facilities and Safety Management resources (Fig. 2B; Table S1).

## QSE: SUPPLIER AND INVENTORY MANAGEMENT

Efficient and cost-effective laboratory operations require the constant availability of quality reagents, supplies, and services. Laboratories should describe agreements regarding the acquisition of equipment, materials, products, and services while also ensuring that all specified criteria for critical supplies and services are consistently met (10, 12). Inventory management, with an adherence to CLIA regulations or applicable regulatory requirements, is paramount in a QMS, whether related to reagents, lots, or storage conditions. However, due to the complexities of NGS (e.g., frequent reagent changes, large numbers of reagent lots to be cataloged and stored, and planning and risk assessments related to changing inventory), satisfying all regulatory criteria can be taxing on the laboratory (11). To aid NGS laboratories in managing inventory, the NGS Quality Initiative has developed three tools related to QSE Supplier and Inventory Management (Table 1; Table S1 [documents 99–101]). The inventory manager is a stand-alone solution that facilitates inventory creation and management, as well as the forecasting of reagent needs. Since 2022, more than 600 downloads have been documented for the QSE Supplier and Inventory Management (Fig. 2B; Table S2).

## QSE: DOCUMENTS AND RECORDS MANAGEMENT

This QSE describes the creation, management, maintenance, control, archival, and retention of documents for the laboratory’s QMS and workflow (12). Laboratory documents and records, whether paper or electronic, must be completed, dated, and populated with correct information to ensure accurate results and patient safety. However, documents and records management can be difficult due to NGS complexity, the large amount of data generated by each test, the format and number of files generated during a sequencing run, and applicable regulatory requirements (Table 1). Record retention times vary based on the regulatory body and accrediting organization. For example, CLIA requires that records be retained for at least 2 years, even in the event of discontinued procedures or methods; this includes emails, method validation plans, and laboratory results, such as sequencing data or QC metrics (11). To help laboratories that perform NGS-based assays, the NGS Quality Initiative has created a Documents and Records Management job aid (Table S1 [document 102]). This tool can help to create documents, establish policies, and determine retention periods according to laboratory needs. Since 2023, there have been nearly 150 downloads of the QSE Documents and Records Management resources (Fig. 2B; Table S2).

## QSE: NONCONFORMING EVENT MANAGEMENT

Nonconforming Event (NCE) Management consists of the requirements for documenting and investigating events that do not conform to the laboratory’s established policies, processes, procedures, or other specified requirements (9, 11). The QSE NCE is critical to mitigate risk and correct deviations from normal practices before larger issues occur. This QSE describes processes to detect and record nonconformances, manage products and services that fail to meet specified requirements, classify the nonconformances for analysis, and correct the problems presented by nonconformances. Due to NGS complexity, laboratories can encounter more frequent NCEs related to personnel, equipment, and process management. The consequences of NCEs can be significant, ranging from minor to critical (e.g., using a sequencer with outdated calibration can lead to incorrect sequencing or misinterpretation that can result in incorrect patient diagnoses and treatments). Adding to the intricacy of this QSE, CLIA requires establishing procedures for monitoring out-of-compliance events (11). To reduce the difficulties associated with this QSE, the NGS Quality Initiative has developed six NCE tools, including procedures for identifying and documenting NCEs, analyzing root causes, and undertaking corrective actions (Table 1; Table S1 [documents 103–108]). Since 2022, there have been over 1,000 downloads of NCE Management resources (Fig. 2B; Table S2).

## QSE: ORGANIZATION AND LEADERSHIP

The QSE Organization and Leadership outlines the criteria for structured roles and responsibilities while also highlighting key leadership functions essential for a laboratory’s success in meeting regulatory and customer requirements (10). Laboratories should have a documented organizational plan and structure that ensures the delivery of services and that details a system-wide approach to quality (12). It is crucial to clearly document the scope of laboratory services, including a description of all testing services provided and the customers served. CLIA requires that all personnel roles, responsibilities, and reporting relationships be documented and communicated effectively (11). This ensures that all staff members are aware of their respective roles within the organization. Poor organization and communication can negatively impact results, final reports, customer satisfaction, and the laboratory’s reputation. To reduce the challenges associated with this QSE, the NGS Quality Initiative has developed three documents with examples of organizational structures and communication plans (Table 1; Table S1 [documents 109–111]). For instance, the NGS Laboratory Communication Plan Guide provides considerations and best practices for conveying essential information to and from leadership, laboratory professionals, internal customers, and external customers. Since 2023, there have been nearly 200 downloads of Organization and Leadership resources (Fig. 2B; Table S2).

## QSE: CUSTOMER FOCUS

The QSE Customer Focus describes the laboratory’s need to identify customers and their expectations, formalize contractual agreements for laboratory services, and manage customer feedback (10, 12). Customers rely on laboratories for critical and highly specialized work, expertise, accurate results, and timely reports, all of which are essential in building and maintaining customer trust and satisfaction. For a laboratory performing NGS, customers can include patients, physicians, genetic counselors, epidemiologists, and others. Failure to meet customers’ expectations may result in delayed decisions and errors, thereby impacting clinical outcomes and/or public health efforts. CLIA requires establishing a system to communicate results and customer feedback during the total testing process (11). Nevertheless, it can be challenging to report NGS results that meet all customer needs. To help laboratories communicate results effectively, the NGS Quality Initiative has created three resources: NGS Result Report Interpretation Guide, NGS Result Report Template, and NGS Training Resource Repository (Table 1; Table S1 [documents 112–114]). Since 2023, there have been over 300 downloads of QSE Customer Focus resources (Fig. 2B; Table S2).

## CONCLUSIONS

Establishing a QMS for laboratories performing NGS is a complex process. Many challenges persist due to the variation in techniques and applications, complexity of the NGS assays, lack of guidance for the development of reference materials, lack of consensus or gold-standard materials for infectious disease testing, and the need for additional-application and agent-specific prescriptive guidance (10, 11, 14, 20, 25). In response, the NGS Quality Initiative has moved beyond producing guidance documents and progressed toward creating practical, implementable tools that address the recurring operational barriers laboratories face when integrating NGS. By mapping resources to the CLSI’s 12 QSEs and providing customizable SOPs, checklists, training materials, validation templates, and bioinformatics toolkits, the Initiative translates high-level quality concepts into concrete workflows and decision aids. These products directly support laboratories in tackling common obstacles (e.g., workforce training, equipment installation and maintenance, data and pipeline documentation, method validation, inventory forecasting, and nonconforming event management), thereby enabling more consistent, reproducible, and auditable NGS practice across diverse settings. The Initiative’s emphasis on usability (e.g., platform-specific pre-installation and maintenance checklists), assessment (e.g., the QMS Assessment Tool), and shared technical solutions helps operationalize quality practices and shorten the time to effective implementation (8). Continued collaboration with federal and nonfederal partners will be essential to refine these tools, expand agent- and application-specific guidance, and advance external quality assurance mechanisms. The NGS Quality Initiative’s practical, QSE-aligned toolkit therefore represents a substantive contribution toward making robust NGS quality management actionable and sustainable in clinical and public health laboratories.

There remains a need to develop and improve methods that continuously promote NGS testing quality, standardization, and implementation while also increasing understanding of test performance (9, 26, 27). Many organizations, both federal and nonfederal, have attempted to satisfy these needs, in part, through the application of prescriptive guidance, regulatory guidelines, and guardrails. Though success has been demonstrated, the need for additional uptake across diverse partners remains. Although the NGS Quality Initiative does not focus on specific international requirements, CLSI’s QSEs can overlap with ISO 15189, which provides users with the potential to customize tools for specific international quality requirements (10).

The NGS Quality Initiative continues to develop tools and resources that close these gaps and address quality-related challenges for NGS laboratories.

## FUTURE DIRECTIONS

Given the increased reliance on sequencing techniques, high-throughput systems will likely become more ubiquitous within public health and clinical settings. At the same time, machine learning and artificial intelligence (AI) are already applied in many laboratories; these tools will continue to improve and grow, and such growth has the potential to greatly benefit those laboratories equipped to harness it. Utilization of machine learning algorithms is already advantageous in clinical practice and can provide enhanced detection and/or identification of disease states, with additional insights into the mechanisms driving disease (28, 29). Other changes could pertain to the evaluation of current applications and the effectiveness of AI in bioinformatics and NGS-related tasks, including research, coding (e.g., pipeline development), and visualization capabilities (28–30). In line with advances in AI, the Initiative is currently exploring natural language processing models to assess method validation plans and increase the efficiency of this process (data not shown).

Advances specific to NGS itself are also expected in the coming years. Along with the rapid growth of NGS-based methods for surveillance and diagnostics comes the requirement for additional standards and further guidance. *In silico* data set standards may be generated according to sequencing-machine parameters and then engineered to include complex genetic traits that might not otherwise be captured in sample cohorts traditionally used to test methods; however, without prescriptive guidance as to the development of gold standards, each laboratory is left to govern the development, quality, and use of data sets. In addition, automated platforms may save time for personnel (e.g., sample-to-result NGS platforms require minimal hands-on tasks), but such approaches often require new training and increased regulatory considerations (31–34).

Going forward, the establishment of NGS assay performance consensus and inter-laboratory comparison methods will be critical. These needs could be addressed, in part, by external quality assurance tools like challenge panels, proficiency testing (PT), or alternative assessment programs, which would allow laboratories to evaluate accuracy and reliability among peers using standardized materials and data files. For example, a Swiss PT study identified differences between bioinformatic workflows and lessons learned to develop future benchmarks for quality control (35). To help address this challenge, the NGS Quality Initiative worked with APHL on a peer network program, in which laboratories can compare workflows on a use-case basis, share sequencing data, develop/deploy new NGS-based QMS practices, and establish collaborations (36).

Additional agent-specific guidelines, gold-standard reference materials, widely available bioinformatic resources, and regulatory guidelines will be needed to standardize the application of NGS. To support this gap, the NGS Quality Initiative collaborated with CDC laboratories and external partners to support use cases for the development and optimization of bioinformatic pipelines, namely, AMR Metagenomics Detection, Simulation, Taxonomic Classification, and Read Optimization and Simulation, Phylogeny Estimation, Read Optimization, Resistance Mutation Identification and Evaluation, and Sequence Annotation. These tools were developed to support laboratories in creating and using *in silico* reference materials by expanding adoption of mutagenized files, evaluating their impact on NGS test development and validation, and providing accessible, reproducible workflows for laboratory generation and use (37, 38).

As the field continues to evolve, the NGS Quality Initiative will continue to connect partners, develop resources, and create new tools to meet current and future public health and clinical laboratory quality needs.

## SUPPLEMENTAL MATERIAL

Supplemental materials jcm.00213-26-s0001.docx Supplemental materials and Tables S1 to S3.

ASM does not own the copyrights to Supplemental Material that may be linked to, or accessed through, an article. The authors have granted ASM a non-exclusive, world-wide license to publish the Supplemental Material files. Please contact the corresponding author directly for reuse.
