# Supplementary material for: The CDC/APHL next generation sequencing quality initiative: practical guidance to implementing quality management systems in laboratories performing next generation sequencing (phase 2)
Source: J Clin Microbiol. 2026 Jul 20;64(8):e00213-26. doi: 10.1128/jcm.00213-26 (PMC13456147; doi:10.1128/jcm.00213-26)
Supplement: Supplementary file 1 [file jcm.00213-26-s0001.docx]

**Supplemental Materials**

**Quality System Procedure:** The Next-Generation Sequencing (NGS) Quality Initiative (QI)’s quality system procedure was previously described (1, 2). In short, the NGS QI collaborates with federal and non-federal partners, including the NGS QI Leadership Team, representatives from the Association of Public Health Laboratories (APHL), members of the Centers for Disease Control and Prevention (CDC), the NGS Quality Workgroup, the Technical Coordinating Committee for the Advanced Molecular Detection (AMD) Platform and NGS QI (TANQ), and partner state and local public health laboratories (PHLs), to address quality-related sequencing challenges. The NGS QI has developed a foundational quality management system (QMS) based on the Clinical and Laboratory Standards Institute's 12 Quality Systems Essentials; this QMS can be integrated into existing laboratory practices or used independently. When gaps are identified, the NGS QI creates new products and resources while ensuring that existing work is not duplicated. Product development begins with approval from the CDC NGS QI Leadership Team and follows a structured review process involving internal and external partners. Each product is overseen by a dedicated document owner and reviewed by subject matter experts. Before public release, all products are made Section 508 compliant and undergo multiple rounds of review. Additionally, published tools are regularly updated through a cyclical review process, typically every three years, with four QSEs targeted for review and gap analysis per year to keep pace with changing standards and policies. NGS QI partners continuously identify quality gaps, seek out existing resources, and develop practical solutions to address emerging challenges (e.g., new platforms).

Once published, NGS QI resources can be sorted by most recently updated or filtered by sequencing platform, QSE, and other criteria. For example, users can select among three NGS quality maturity levels: Emerging (laboratories with no or limited quality management in place or those developing a QMS), Progressing (laboratories with externally regulated activities but critical gaps), and Established (laboratories with a monitored and sustainable QMS that meets regulatory requirements).

The Emerging category filters resources intended as a starter toolkit, including assessment tools and user guides, validation templates (e.g., NGS method validation plan, SOP, and summary report), pre-installation and equipment checklists for multiple sequencers, bioinformatics documentation and containerization guidance, training SOPs, and starter inventory and records templates.

At the Progressing maturity level, laboratories move beyond ad hoc practices to implement foundational quality management elements. Key processes are documented, basic competency training is established, and critical equipment and supplies are controlled. Quality metrics and corrective-action procedures are collected and applied; however, their use for routine trend analysis and proactive risk mitigation is inconsistent, limiting full reproducibility and optimization across workflows.

Within the Established maturity level, laboratories maintain a comprehensive and consistently implemented QMS, which includes standardized SOPs, routine validation and verification practices, and documented competency programs for all relevant personnel. Performance metrics are regularly analyzed to support proactive risk management and continuous improvement, resulting in reliable, reproducible assay performance across workflows.

Although the NGS QI does not provide direct support to international users, the Initiative may provide indirect technical support through U.S. partners who collaborate with international laboratories.

1.	Hutchins RJ, Phan KL, Saboor A, Miller JD, Muehlenbachs A. 2019. Practical Guidance to Implementing Quality Management Systems in Public Health Laboratories Performing Next-Generation Sequencing: Personnel, Equipment, and Process Management (Phase 1). J Clin Microbiol 57.

2.	Cherney B, Diaz A, Chavis C, Ghattas C, Evans D, Arambula D, Stang H. 2025. The Next-Generation Sequencing Quality Initiative and Challenges in Clinical and Public Health Laboratories. Emerg Infect Dis 31.

**Table S1.** [**https://www.cdc.gov/lab-quality/php/ngs-quality-initiative/qms-tools-resources.html**](https://www.cdc.gov/lab-quality/php/ngs-quality-initiative/qms-tools-resources.html). Numbers are paired with numbers inside Table 1.

**QSE – Personnel Management**

1. Bioinformatics Employee Training SOP
2. CLC Genomics Workbench Employee Training Form
3. *De novo* Genome Assembly Employee Training Form
4. Ion PGM Sequencer Competency Assessment Form
5. Ion PGM Sequencer Competency Assessment SOP
6. Ion PGM Sequencer Employee Training Form
7. Ion PGM Sequencer Employee Training SOP
8. Ion PGM Sequencer Trainer Designation Form
9. iSeq 100 Sequencer Employee Training Form
10. iSeq 100 Sequencer Employee Training SOP
11. MinION Sequencer Employee Training Form
12. MinION Sequencer Employee Training SOP
13. MiniSeq Sequencer Employee Training Form
14. MiniSeq Sequencer Employee Training SOP
15. MiSeq Sequencer Competency Assessment Form
16. MiSeq Sequencer Competency Assessment SOP
17. MiSeq Sequencer Employee Training Form
18. MiSeq Sequencer Employee Training SOP
19. MiSeq Sequencer Trainer Designation Form
20. NextSeq 500/550 Sequencer Employee Training Form
21. NextSeq 500/550 Sequencer Employee Training SOP
22. Operating in Linux Environment Employee Training Form
23. Preprocessing Read Quality Control Employee Training Form
24. Read Trimming of Next Generation Sequencing Data Training Form
25. Short Read Alignment Employee Training Form

**QSE – Equipment Management**

26. Error Log for Illumina Sequencers
27. Error Log for Thermo Fisher Sequencers
28. Ion Chef Sequencer In-Use Maintenance Log
29. Ion Chef Sequencer Maintenance SOP
30. Ion OneTouch 2 Sequencer In-Use Maintenance Log
31. Ion OneTouch 2 Sequencer Maintenance SOP
32. Ion OneTouch ES Sequencer In-Use Maintenance Log
33. Ion OneTouch ES Sequencer Maintenance SOP
34. Ion PGM Sequencer In-Use Daily Maintenance Log
35. Ion PGM Sequencer In-Use Weekly Maintenance Log
36. Ion PGM Sequencer Maintenance SOP
37. Ion PGM Sequencer Maintenance Wash Flowchart
38. Ion PGM Sequencer Power-Off Maintenance Log
39. Ion PGM Sequencer Pre-Installation Checklist
40. Ion PGM Sequencer Software Update Evaluation SOP
41. Ion PGM Sequencer Software Update Form
42. iSeq 100 Sequencer In-Use Maintenance Log
43. iSeq 100 Sequencer Maintenance SOP
44. iSeq 100 Sequencer Pre-Installation Checklist
45. Key Criteria for Selection of Liquid Handlers for NGS
46. Key Criteria for Selection of NGS Equipment
47. MinION Mk1B Sequencer Pre-Installation Checklist
48. MiniSeq Sequencer In-Use Maintenance Log
49. MiniSeq Sequencer Maintenance SOP
50. MiniSeq Sequencer Pre-Installation Checklist
51. MiSeq Sequencer In-Use Maintenance Log
52. MiSeq Sequencer Maintenance SOP
53. MiSeq Sequencer Maintenance Wash Flowchart
54. MiSeq Sequencer Pre-Installation Checklist
55. MiSeq Sequencer Software Update Evaluation SOP
56. MiSeq Sequencer Software Update Form
57. MiSeq Sequencer Standby Maintenance Log
58. NextSeq 500/550 Sequencer In-Use Maintenance Log
59. NextSeq 500/550 Sequencer Maintenance SOP
60. NextSeq 500/550 Sequencer Pre-Installation Checklist
61. Vendor-Performed IQ-OQ Coversheet

**QSE – Process Management**

62. Bioinformatics QC: Assembly Quality Control Guidance
63. Bioinformatics Analysis QC Guidance for NGS Workflows
64. Individualized Quality Control Plan (IQCP) Template
65. NGS Method Validation Plan Template
66. NGS Method Validation SOP
67. NGS Method Validation Summary Report Template
68. NGS MinION 1D QC Workflows Guidance
69. NGS MinION Rapid QC Workflows Guidance
70. NGS QC Guidance for Illumina Workflows
71. Bioinformatics QC: Pre-Analysis Quality Control (FASTQ) Procedure
72. SanitizeME: Host DNA Removal SOP
73. Sequencing QC: Quality Control and Raw Illumina Data

**QSE – Information Management**

74. Bioinformatics Considerations for Pipeline Planning and Development
75. Bioinformatics Pipeline Containerization Purpose and Approaches
76. Bioinformatics Pipeline Documentation Standardization – Laboratory Management Briefing
77. Bioinformatics Pipeline Documentation Standardization – Technical Best Practices
78. Bioinformatics Validation Plan Template
79. Building an In-House Reference Database
80. Docker Container Requirements, Installation Execution SOP
81. Downloading Data from Public DNA Sequence Reference Database
82. File Integrity Check SHA-2 Checksum
83. Package and Environment Management Systems for Bioinformatics Briefing
84. Sequencing Data Retention and Storage Best Practices
85. Singularity Overview Briefing
86. Uploading Data to Public Reference Databases
87. Visualization Product

**QSE – Assessments**

88. Bioinformatician Competency Assessment Form
89. Bioinformatician Competency Assessment SOP
90. Proficiency Testing and Alternative Assessment
91. QMS Assessment Tool

**QSE – Continual Improvement**

92. Identifying and Monitoring NGS Key Performance Indicators SOP
93. Key Performance Indicator Development Form

**QSE – Facilities and Safety Management**

94. Illumina iSeq 100 Waste Disposal SOP
95. Illumina MiniSeq Waste Disposal SOP
96. Illumina MiSeq Waste Disposal SOP
97. Illumina NextSeq 500/550 Waste Disposal SOP
98. Oxford Nanopore MinION Flow Cell Return Disposal Job Aid

**QSE – Supplier and Inventory Management**

99. Inventory Management Database User Guide
100. Inventory Manager Tool
101. Summary of Inventory Management Processes

**QSE – Documents and Records Management**

102. Documents and Records Management Job Aid

**QSE – Nonconforming Event Management**

103. Corrective Action/Preventive Action Procedure
104. Identifying and Documenting Nonconforming Events
105. Nonconforming Event Report Closeout and Trending
106. Nonconforming Event Types and Common Root Causes
107. Root Cause Analysis (RCA) Job Aid
108. Root Cause Analysis Procedure

**QSE – Organization and Leadership**

109. NGS Implementation Communication Plan Example Template
110. NGS Implementation Lessons Learned for Organizational Structure
111. NGS Laboratory Communication Plan Guide

**QSE – Customer Focus**

112. NGS Result Report Interpretation Guide
113. NGS Result Report Template
114. NGS Trainings Resource Repository

**Table S2.** Total number of visits to the NGS Qualitative Initiative website by state in the U.S. from 2020 (Quarter 1) to 2025 (Quarter 2) was 47,699.

| **State** | **Visits** |  | **State** | **Visits** |  | **State** | **Visits** |
| --- | --- | --- | --- | --- | --- | --- | --- |
| AK | 201 |  | KY | 263 |  | NY | 2485 |
| AL | 252 |  | LA | 260 |  | OH | 1075 |
| AR | 93 |  | MA | 1167 |  | OK | 163 |
| AZ | 570 |  | MD | 1714 |  | OR | 320 |
| CA | 3875 |  | ME | 168 |  | PA | 1127 |
| CO | 854 |  | MI | 634 |  | RI | 82 |
| CT | 307 |  | MN | 747 |  | SC | 1033 |
| DC | 506 |  | MO | 243 |  | SD | 42 |
| DE | 314 |  | MS | 104 |  | TN | 543 |
| FL | 1305 |  | MT | 71 |  | TX | 2423 |
| GA | 14553 |  | NC | 1085 |  | UT | 346 |
| HI | 273 |  | ND | 41 |  | VA | 3057 |
| IA | 348 |  | NE | 99 |  | VT | 54 |
| ID | 108 |  | NH | 113 |  | WA | 1010 |
| IL | 1029 |  | NJ | 790 |  | WI | 604 |
| IN | 451 |  | NM | 136 |  | WV | 150 |
| KS | 129 |  | NV | 132 |  | WY | 250 |

**Table S3.** Total downloads of NGS Quality Initiative tools per Quality System Essential across the U.S. from 2020 (Quarter 1) to 2025 (Quarter 2) was 29,051. Detection of downloads by location was unavailable.

| **Year** | **Personnel**  **Management** | **Equipment**  **Management** | **Process**  **Management** | **Information**  **Management** | **Assessments** | **Continual Improvement** |
| --- | --- | --- | --- | --- | --- | --- |
| 2025 | 0 | 0 | 741 | 400 | 347 | 195 |
| 2024 | 1146 | 1241 | 1950 | 1095 | 1132 | 684 |
| 2023 | 1021 | 1097 | 1236 | 470 | 760 | 420 |
| 2022 | 946 | 973 | 1205 | 479 | 621 | 564 |
| 2021 | 928 | 894 | 1297 | 550 | 563 | 602 |
| 2020 | 460 | 743 | 738 | 112 | 194 | 145 |

| **Year** | **Facilities and Safety**  **Management** | **Supplier and Inventory Management** | **Documents and Records**  **Management** | **Nonconforming**  **Event Management** | **Organization and Leadership** | **Customer Focus** |
| --- | --- | --- | --- | --- | --- | --- |
| 2025 | 0 | 124 | 0 | 198 | 0 | 101 |
| 2024 | 120 | 238 | 112 | 502 | 150 | 196 |
| 2023 | 130 | 155 | 32 | 295 | 49 | 73 |
| 2022 | 213 | 90 | 0 | 50 | 0 | 0 |
| 2021 | 191 | 0 | 0 | 0 | 0 | 0 |
| 2020 | 83 | 0 | 0 | 0 | 0 | 0 |
